# Patient-guided modifications of oral anticoagulant drug intake during Ramadan fasting: a multicenter cross-sectional study

**DOI:** 10.1007/s11239-020-02218-0

**Published:** 2020-07-14

**Authors:** AbdulAziz Batarfi, Haitham Alenezi, Abdulrahman Alshehri, Saud Balelah, Hameedullah Kazim, Mohammed Algthami, Mariam M. Hussain, Nada Alshehri, Rahaf Alsharif, Hadeel Ashour, Mutaz Althobaiti, Shomokh Alotaibi, Helmuth Steinmetz, Christian Foerch

**Affiliations:** 1 Department of Neurology, University Hospital of Frankfurt, Goethe-University, Schleusenweg 2-16, 60528 Frankfurt am Main, Germany; 2 Department of Cardiology, King AbdulAziz Medical City Complex, Riyadh, Saudi Arabia; 3 Department of Internal Medicine, Aseer Central Hospital, Abha, Saudi Arabia; 4 Department of Internal Medicine, King Fahad General Hospital, Madinah, Saudi Arabia; 5 Department of Cardiology, Al Hada Armed Forces Hospital, Taif, Saudi Arabia; 6 Department of Internal Medicine, Al Hada Armed Forces Hospital, Taif, Saudi Arabia; 7 College of Medicine, King Saud Bin AbdulAziz University, Riyadh, Saudi Arabia; 8 College of Medicine, King Khaled University, Abha, Saudi Arabia; 9 College of Medicine, Taibah University, Madinah, Saudi Arabia; 10 College of Medicine, Taif University, Taif, Saudi Arabia; 11 College of Medicine, King AbdulAziz University, Jeddah, Saudi Arabia

**Keywords:** Oral anticoagulation, Ramadan, Fasting, Saudi Arabia, Adherence, Education

## Abstract

Fasting Ramadan is known to influence patients’ medication adherence. Data on patients’ behavior to oral anticoagulant (OAC) drug intake during Ramadan is missing. We aimed to determine patient-guided modifications of OAC medication regimen during Ramadan and to evaluate its consequences. A multicenter cross-sectional study conducted in Saudi Arabia. Data were collected shortly after Ramadan 2019. Participants were patients who fasted Ramadan and who were on long-term anticoagulation. Patient-guided medication changes during Ramadan in comparison to the regular intake schedule before Ramadan were recorded. Modification behavior was compared between twice daily (BID) and once daily (QD) treatment regimens. Rates of hospital admission during Ramadan were determined. We included 808 patients. During Ramadan, 53.1% modified their intake schedule (31.1% adjusted intake time, 13.2% skipped intakes, 2.2% took double dosing). A higher frequency of patient-guided modification was observed in patients on BID regimen compared to QD regimen. During Ramadan, 11.3% of patients were admitted to hospital. Patient-guided modification was a strong predictor for hospital admission. Patient-guided modification of OAC intake during Ramadan is common, particularly in patients on BID regimen. It increases the risk of hospital admission during Ramadan. Planning of OAC intake during Ramadan and patient education on the risk of low adherence are advisable.

**Electronic supplementary material:**

The online version of this article (10.1007/s11239-020-02218-0) contains supplementary material, which is available to authorized users.

## Highlights

- Patient-guided modification of OAC regimen is common during Ramadan.
- Fasting patients on BID regimen face a conflict in taking the morning dose as prescribed to them, and therefore, Patient-guided modification are more in this group.
- The chance of hospital admission during Ramadan triples in patients who modify their regimen.
- Patient education and modifying the intake of OAC prior to Ramadan to suit fasting times is of an essence.

## Introduction

One of the most important events in the Islamic calendar is the act of fasting during Ramadan. Around 1.6 billion Muslims practice this community celebration. All adult Muslims are required to refrain from taking food and beverages between the beginning of the morning twilight (Fajr) and sunset. This includes the oral intake of medications [1, 2]. Exceptions are granted to pregnant and lactating women, sick people, and elderly who cannot tolerate the fasting [3]. A conflict, however, exists for patients with chronic diseases and for individuals who are on regular prophylactic drug treatment [4–7].

Patient-guided modification of medication regimen during Ramadan is common. Aslam et. al. found that only 42% of the surveyed 81 Asian Muslim patients were adherent to their usual treatment schedule during Ramadan. The remaining changed their intake pattern and skipped doses or took them at different timings [8]. In another survey conducted in Kuwait, 64% of the patients were found to alter their treatment schemes during Ramadan, many of them taking their tablets as a single dose instead of divided (i.e. double dosing). Particularly in elderly patients, this led to potentially serious side-effects [9]. The clinical importance of patient-guided modification during Ramadan was reported in other studies as well [10, 11].

For oral anticoagulant drug (OAC) treatment, the patient-guided modification of medication regimen appears to be particularly critical, both in terms of thrombotic and bleeding complications. However, a prospective evaluation of this behavior in the context of OAC (including warfarin and direct oral anticoagulants [DOAC]) during Ramadan has never been performed. What is known from a real world systematic review comprising 1.6 million patients with atrial fibrillation is that overall adherence to DOAC (irrespective of Ramadan) is relatively low. Moreover, the study highlighted suboptimal adherence to DOAC as a risk factor affecting clinical outcomes, with higher rate of non-adherent patients having bleeding events [12].

In the current study we aimed to characterize patient-guided modification of OAC intake during Ramadan. We hypothesized that OAC medication that has to be taken twice daily (BID) is associated with higher rates of self-guided modification than once daily (QD) medication. We also determined the risk of complications resulting from patient-guided modification of treatment regimen.

## Methods

We designed a multi-center cross-sectional study targeted to examine the behavior of fasting Muslims to OAC intake during Ramadan. A questionnaire was created for the purpose of data collection. The data collection was obtained through face-to-face interviews from trained medical personnel at regular patient visits in different outpatient clinics (neurology, hematology, cardiology departments, and anticoagulation clinics) in six centers distributed around Saudi Arabia (King Abdulaziz University Hospital, Jeddah; King Fahad General Hospital, Madinah; King Faisal Medical Center, Taif; Al Hada Military Hospital, Taif; Aseer Central Hospital, Abha; and King Abdulaziz Medical City Complex, Riyadh). In 2019, Ramadan lasted from May 6th to June 4th. The data were collected in July and August 2019 to ensure full representation of data through the whole month. For the capital city (Riyadh) on June 3rd, for example, the beginning of morning twilight (Fajr, beginning of fasting) was at 3:52 am and the sunset (end of fasting) was at 6:39 pm, resulting in a fasting time of 14 h and 47 min.

This study was performed in accordance with the declaration of Helsinki. Ethical approval was obtained from the ethical committee of each participating center. All subjects included in this study gave informed consent prior to study inclusion.

Inclusion criteria were (i) age ≥ 18 years, (ii) Muslim religion with active fasting during Ramadan in 2019, (iii) long-term anticoagulant treatment (> 3 months) with warfarin or DOAC (including apixaban, dabigatran, edoxaban, rivaroxaban). Patients who fasted only partially during Ramadan and patients who were not fit to fast were excluded. Patients who switched their anticoagulation therapy during the study period were also excluded. The focus of the study was on evaluating oral anticoagulation drugs and its intake frequency (QD vs. BID) during Ramadan. Therefore, patients on other types of antithrombotic medication (e.g. aspirin, clopidogrel, heparin) were not evaluated.

In Saudi Arabia, all Saudi nationality patients are fully covered by the governmental health system. Non-Saudi nationality patients requires private insurance coverage in order to be treated in Saudi Arabia, unless presented as emergent case. Warfarin and DOAC are nationally approved in Saudi Arabia. Patient information leaflets were evaluated for the recommended intake regimen of all five anticoagulants (warfarin: QD only, dabigatran: BID only, apixaban: BID only, rivaroxaban: QD only, and edoxaban: QD only) [13–15].

Different variables were collected through the questionnaire (see supplemental materials). This includes study site, patients’ age and sex, indication for OAC, and OAC dose and intake schedule (as prescribed by the responsible physician). Patient-guided changes of OAC intake during Ramadan were recorded in comparison to the regular intake schedule (i.e. the last prescribed medication plan) before and after Ramadan. This included (I) adjustment in the time of intake, (II) changes in the number of intake (skipping doses), and (III) double dosing. Reporting hospital admission during Ramadan and reasons for admission were as well recorded and evaluated.

### Statistical analysis

Sample size calculation was performed using an online tool (www.clincalc.com). Based on observational data and marketing analyses [16–18], we assumed the share of OAC medication in Saudi Arabia to be as follows: warfarin 50%, apixaban 15%, rivaroxaban 15%, dabigatran 15%, edoxaban 5% (i.e. 30% BID and 70% QD). Based on studies that reported a reduction of at least one third in medication adherence during Ramadan in comparison to regular times [7, 19] and others that pointed out the unsuitability of using BID doses during Ramadan in comparison to QD due to noncompliance [20], we assumed that adherence during Ramadan compared to the regular intake schedule is 75% in patients on QD medication, with a 20% reduction in adherence in patients on BID medication. With a power of 80% and an alpha error of 5%, the minimum estimated sample size was 152 subjects for each group (BID vs. QD). However, a larger sample size has been achieved at the end of the study period.

Data analyses were made using Statistical Package for the Social Sciences software (SPSS) version 26. Descriptive statistics were used to report patient demographics. Continuous data were presented as mean (± SD), categorical data were presented as median (interquartile range—IQR). Chi square test was used to compare medication adherence between patients taking BID and QD medication, respectively. Binary multivariate analysis was performed to identify independent predictors for patient-guided modification of treatment schedules during Ramadan. A second binary multivariate analysis was performed to identify independent predictors of hospital admission during Ramadan.

## Results

Questionnaires from 813 subjects were collected, but only 809 fulfilled all inclusion criteria. The remaining 4 subjects were under the age of 18 (n = 3) or did not fill in the required information (n = 1) and were therefore excluded from the analysis. One subject switched from edoxaban to warfarin during Ramadan and was also excluded. The remaining 808 subjects had a mean (± standard deviation [SD]) age of 56.3 (13.8) years (range 18–96 years). Females comprised 57.4% (n = 464) of the sample, and Saudis constituted 98.6% (n = 797) of the sample. The proportion of subjects who had a bachelor degree or higher were 26% (n = 210) (Table 1). The share of the data collection between centers was as follows: King Faisal Medical Center (n = 250, 30.9%), King Abdullah General Hospital (n = 216, 26.7%), Aseer Central Hospital (n = 162, 20.0%), King Abdulaziz University Hospital (n = 10,1 .2%), King Abdulaziz Medical City (n = 79, 9.8%), and Al Hada Armed Forces (n = 91,11. 3%). The share of data collection between outpatient clinics was as follows: Anticoagulation clinics (n = 312, 38.6%), cardiology (n = 350, 43.3%), hematology (n = 65, 8.0%), and neurology (n = 81, 10.0%).

**Table 1:** General comparison between each direct oral anticoagulation drug and Vitamin-K antagonist

	Warfarin	Apixaban	Dabigatran	Edoxaban	Rivaroxaban	Total
n (%)	553 (68.4)	130 (16.1)	58 (7.2)	4 (0.5)	63 (7.8)	808 (100)
Mean age (+/− SD)	54.7 (13.7)	61.2 (12.4)	56.9 (12.5)	48.3 (18.9)	60.2 (15.7)	56.3 (13.8)
Females, n (%)	331 (59.9)	78 (60.0)	20 (34.5)	4 (100)	31 (49.2)	464 (57.4)
Saudi nationality, n (%)	544 (98.4)	130 (100)	57 (98.3)	4 (100)	62 (98.4)	797 (98.6)
Bachelor degree or higher, n (%)	144 (26.0)	27 (20.8)	25 (43.1)	1 (25.0)	13 (20.6)	210 (26.0)
Indication for anticoagulation intake, n (%)						
• Heart valve disease	252 (45.6)	13 (10.0)	20 (34.5)	0 (0.0)	7 (11.1)	292 (36.1)
• Vein thrombosis	104 (18.8)	18 (13.8)	9 (15.5)	3 (75.0)	17 (27.0)	151 (18.7)
• Heart rhythm disease	40 (7.2)	56 (43.1)	15 (25.9)	0 (0.0)	13 (20.6)	124 (15.3)
• Other cardiological diseases^a^	47 (8.5)	21 (16.2)	7 (12.1)	1 (25.0)	10 (15.9)	86 (10.6)
• Stroke	52 (9.4)	13 (10.0)	3 (5.2)	0 (0.0)	12 (19.0)	80 (9.9)
• Pulmonary diseases^b^	22 (4.0)	4 (3.1)	1 (1.7)	0 (0.0)	0 (0.0)	27 (3.3)
• Multiple indications	16 (2.9)	1 (0.8)	0 (0.0)	0 (0.0)	0 (0.0)	17 (2.1)
• Other indications^c^	18 (3.3)	3 (2.3)	2 (3.4)	0 (0.0)	3 (4.8)	26 (3.2)
• Not known	2 (0.4)	1 (0.8)	1 (1.7)	0 (0.0)	1 (1.6)	5 (0.6)
Patients on BID regimen, n (%)	37 (6.7)	80 (61.5)	18 (31.0)	0 (0.0)	19 (30.2)	154 (19.1)
Share of typical anticoagulation dosages, mg (n; %)	3 (96; 17.4) 6 (29; 5.2)	2.5 (50; 38.5) 5 (71; 54.6)	110 (4; 6.9) 150 (24; 41.4)	30 (3; 75) 60 (1; 25)	10 (27; 42.9) 20 (14; 22.2)	

^a^Other cardiological diseases including: coronary heart disease, previous myocardial infarctions and cardiomyopathies

^b^Pulmonary diseases including: pulmonary embolism and pulmonary artery hypertension

^c^Other indications including: antiphospholipid syndrome and other coagulation disorders

About two thirds of the subjects included in the study were anticoagulated with warfarin (68.4%, n = 553), while the remaining one third (31.6%, n = 255) was treated with DOACs (16.1% [of all patients] with apixaban, 7.2% with dabigatran, 0.5% with edoxaban, and 7.8% with rivaroxaban) (Table 1). Around one fifth (19.1%) of the patients were prescribed to take their anticoagulation medication as BID. This includes 6.7% of warfarin users, 31% of dabigatran users (although only BID is approved), 61.5% of apixaban users and 30.2% of rivaroxaban users (although only QD is approved). All edoxaban users (n = 4) were prescribed to take their anticoagulant QD (Table 1). Most common indication for OAC was heart valve disease (36.1%, n = 292) followed by vein thrombosis (18.7%, n = 151) and heart rhythm disease (15.3%, n = 124).

**Fig. 1:**
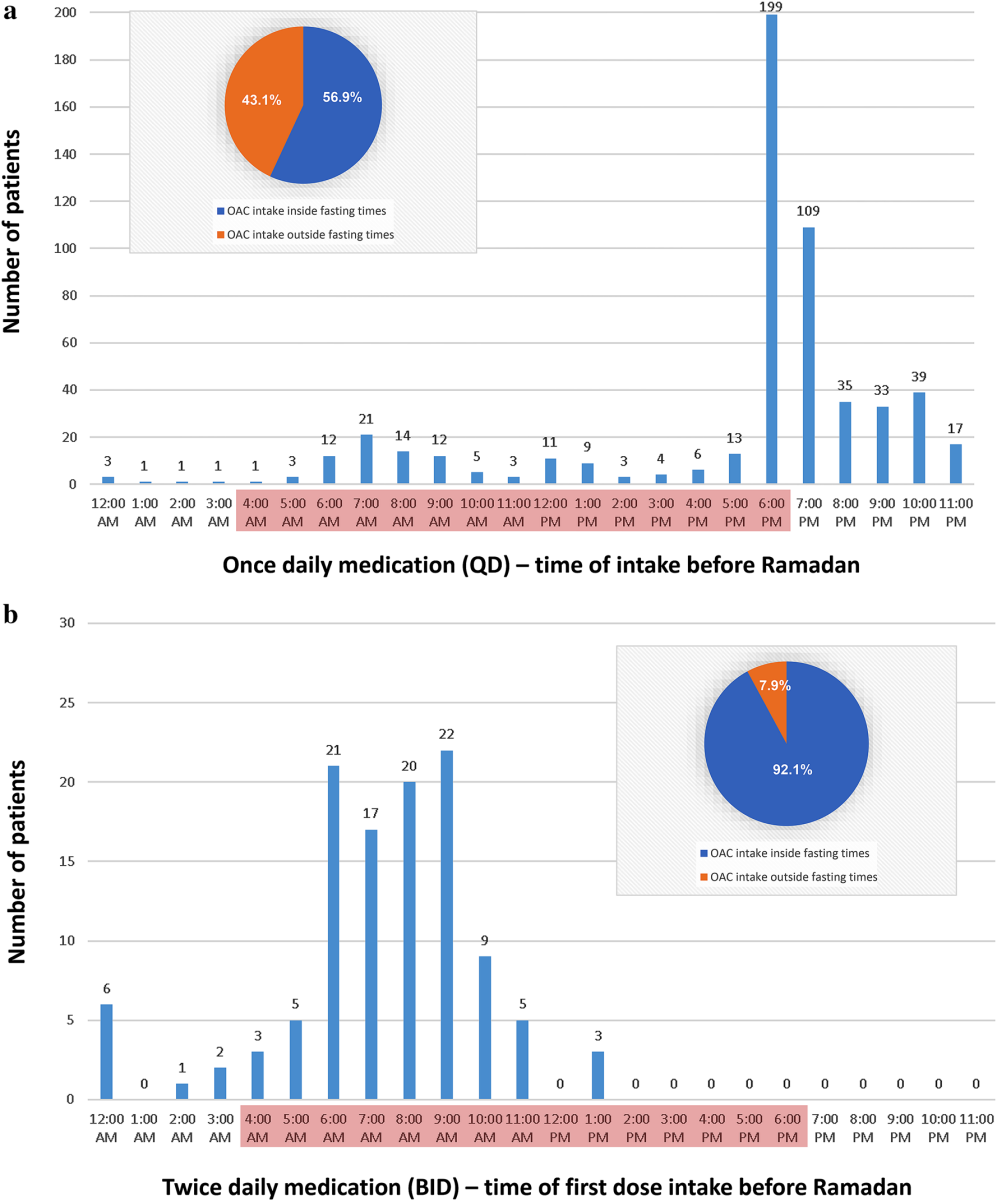

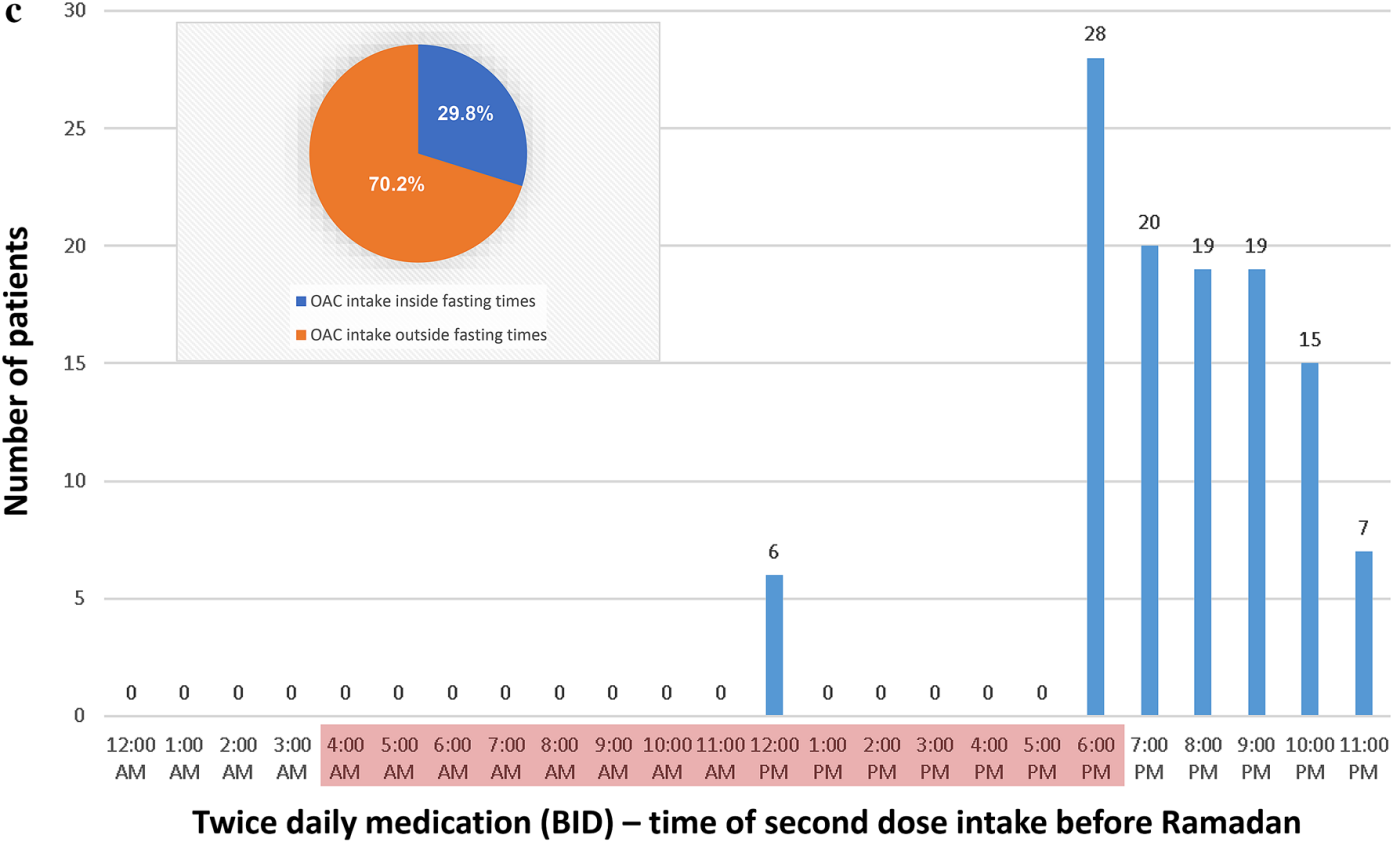
Schedule of OAC intake during regular times (before Ramadan) for patients taking OAC once daily (**a**), or twice daily (first intake [**b**] and second intake [**c**]). The red mark indicates the daily Ramadan fasting period (for Riyadh on June 3rd, 2019: Fajr [beginning of fasting] 3:52 am, sunset [end of fasting] 6:39 pm). A massive conflict with fasting times arises for the first dose of BID anticoagulant medication. The circle diagram depicts the proportion of OAC intakes that lay in (later on) Ramadan fasting times

Before Ramadan, most of the patients who were prescribed to take their anticoagulation drugs once daily (QD) were taking their medication at 6 pm (35.8%). Only a minority was taking their medication in the morning or late evening. In contrast, more than the half of the patients who were prescribed to take their anticoagulation twice daily (BID) had their first intake between 6 and 9 am. Notably, for the vast majority of BID patients (92.1%), the time of the first dose intake lies within the fasting period (Fig. 1).

During Ramadan, 46.9% (n = 379) of patients reported taking OAC medication as prescribed to them. The remaining 53.1% (n = 429) reported to have modified drug intake in different ways to suit their fasting times (31.1% reported adjusting the time of intake, 13.2% skipped intakes, and 2.2% reported double dosing). More patients on BID anticoagulation (73.4%) have modified their medication plan than patients on QD anticoagulation (48.3%) (p < 0.001). Similarly, more patients on BID anticoagulation changed the time of intake (57.8% vs. 24.8%; p < 0.001), skipped doses (28.6% vs. 9.6%; p < 0.001), or took double dosing of their anticoagulant (11.0% vs. 0.2%; p < 0.001) than patients on QD anticoagulation. In a multivariable analysis with patient-guided modification of OAC as dependent variable and age, gender, nationality, educational background and the number of intake per day as independent variables, BID was a strong independent predictor for modification during Ramadan (OR 2.911; 95% CI 1.964–4.314; p < 0.001) (Table 2). Low educational background (less than bachelor degree) was identified as another independent predictor of patient-guided therapy modification during Ramadan (p = 0.004).

**Table 2:** Multivariable analysis with correlation’s significance and odd ratio

Independent variable	Dependent variable	Odd ratio	95% Confidence coefficient		P-value
			Lower limit	Upper limit	
Age	Modified plan (vs. as prescribed)	1.001	0.990	1.012	0.882
Female (vs. male)	Modified plan (vs. as prescribed)	0.869	0.646	1.170	0.356
Saudi nationality (vs. others)	Modified plan (vs. as prescribed)	1.874	0.541	6.486	0.322
High educational background (vs. low)	Modified plan (vs. as prescribed)	0.603	0.426	0.853	0.004
BID (vs. QD)	Modified plan (vs. as prescribed)	2.911	1.964	4.314	< 0.001
Age	Admitted in hospital (vs. not admitted)	0.997	0.981	1.013	0.697
Female (vs. male)	Admitted in hospital (vs. not admitted)	1.597	0.987	2.583	0.057
Saudi nationality (vs. others)	Admitted in hospital (vs. not admitted)	0.535	0.109	2.619	0.440
High educational background (vs. low)	Admitted in hospital (vs. not admitted)	0.695	0.380	1.272	0.238
BID (vs. QD)	Admitted in hospital (vs. not admitted)	0.817	0.458	1.459	0.495
Modified plan (vs. as prescribed)	Admitted in hospital (vs. not admitted)	2.660	1.622	4.360	< 0.001

From the total included patients, 11.3% (n = 91) were admitted to a hospital during Ramadan. Cardiac diseases were the most common reason, but bleeding complications (n = 16) and abnormal coagulation profiles (n = 7) were also frequently reported (Table 3). The rate of hospital admission was higher in patients that modified treatment regimes (15.4%) as compared to adherent patients (6.6%). In a multivariable analysis with hospital admission during Ramadan as dependent variable and age, gender, nationality, educational background, the number of intake per day, and patient-guided modification of OAC plan during Ramadan as independent variables, patient-guided modification was a strong independent predictor for hospital admission during Ramadan (OR 2.660; 95% CI 1.622–4.360; p < 0.001) (Table 2).

**Table 3:** Number and percentage of the reasons of hospital admission during the month of Ramadan

Reason of hospital admission	Number of subjects (n)	Percent (%)
Cardiological issues^a^	24	27.9
Pulmonary embolism	5	5.8
Thrombosis	6	7.0
Stroke or TIA	5	5.8
Abnormal or non-therapeutic coagulation profile	7	8.1
Bleeding, bruises or anemia	16	18.6
Multiple reasons	2	2.3
Other reasons	21	24.4

^a^Cardiological issues including: cardiac arrythmia, heart failure, myocardial infarction, coronary heart diseases and others

## Discussion

Our analysis demonstrated that patient-guided modification of OAC treatment regimen is common during Ramadan. Fasting patients on BID medication encounter a massive conflict in taking the morning dose as prescribed. This leads to significant changes in OAC regimen, particularly in BID patients. In consequence, the chance of being admitted to hospital during Ramadan almost triples for patients undertaking self-guided modification of OAC treatment regimen.

The current study was performed in Saudi Arabia, where Ramadan is the largest Muslim celebration of the year. A strong desire in the community (even for patients granted exceptions) to participate in all aspects of this celebration, including fasting, is well known [5–7, 21]. In this study, warfarin was more commonly used than DOAC. This is different from what is reported worldwide in regard to anticoagulant usage. It is notable, however, that DOAC is being increasingly used in the clinical practice both in Saudi Arabia and the Middle East [18]. Even though the general adherence to DOAC, irrespective to Ramadan, is reported as suboptimal worldwide [12], the current study showed a remarkably low adherence in terms of approval-conform intake even before the beginning of Ramadan. Many DOAC users (particularly those treated with dabigatran) were prescribed their anticoagulant inappropriately, resulting in under-dosing. The fear for bleeding complications is a likely cause for this finding. Other studies also reported inappropriate prescription of DOAC in Saudi Arabia, including under-dosing or non-approved indications [13, 15, 22, 23]. This finding points to the importance of ensuring an approval-conform intake of OAC medication in general.

Our study analyzed the time of anticoagulation intake in detail. We identified a massive conflict for BID patients during Ramadan, when 92% of the regular morning doses fall within fasting time. Patients are forced to make wide adjustments of their time of intake to suit their fasting. On the other hand, patients prescribed OAC as QD were mainly taking their anticoagulant at 6 pm before Ramadan. Even though the time of intake still lies within fasting period (ending around 6:40 pm in Riyadh in 2019), delaying the intake around 1 h during Ramadan raises lesser problems.

Analyzing patients’ behavior of OAC intake during Ramadan in more detail showed that more than the half reported modifying their treatment plan during Ramadan without prior physician’s consultation (including change of intake times, dose skipping, and taking both doses at the same time). Taking tablets as BID was a strong predictor of patient-guided modification of treatment during Ramadan (regardless of the type of OAC being used). Additionally, low educational background was also identified as a predictor of reduced adherence during Ramadan [21, 24–26].

Patient-guided modifications of OAC therapy can lead to under- and overdosing [27–29]. This in turn increases the risk of complications. In consequence, the chance of being admitted to hospital during Ramadan almost tripled for patients undertaking self-guided modification. Reasons for admission were related to anticoagulation over- and underdosing in many of the cases.

It is therefore imperative that patients follow approval-conform intake schedules even during Ramadan. Our data support the available literature pointing to the importance of pre-Ramadan education [24–26] and we recommend patient encouragement for adherence. Possible complications in case of noncompliance or self-modification of intake regimen should also be discussed. In case of anticipated conflicts between treatment regimen and fasting times physicians may adjust intake times to what is meaningful and justifiable from a medical perspective. For BID patients, an adjustment of the time of intake of both doses with special consideration on both fasting time and a sufficient time gap between the intakes seems possible [2, 21]. Some authors suggested the possibility of switching from multiple doses medication to long-acting/sustained release QD medication during Ramadan (e.g. for Parkinson’s disease) [21, 30]. Whether it is reasonable to switch OAC patients from BID medication to QD medication appears however questionable. Many other factors determine the selection of the “optimal” DOAC medication for the individual patient. Islam permits the breaking of fasting for those suffering from illnesses when fasting may interfere with their health or harm them in a way. According to the holy Qur’an, these individuals are allowed to either make up for those days in other time of the year when they are able to fast (for example in winter when fasting periods are shorter) or they get redemption by feeding a poor person. Discussing these options with the patient is another possible approach for managing OAC during Ramadan [2].

### Limitations

This study was conducted in Saudi Arabia. The generalizability of the results to all Muslim communities worldwide may be limited, as cultural influences could also play a role in patient’s behavior during Ramadan. The effects of the suboptimal adherence prior to the beginning of Ramadan have not been examined, although this could also cause increased risks as compared to patients with optimal compliance (regardless of fasting during Ramadan). Hence, future evaluations seem to be mandatory in order to reduce the risk resulting from reduced adherence both within and outside Ramadan. The missing information regarding the effect of insurance coverage on medication intake add a limitation to the study, although the proportion of the non-Saudi nationality patients was very low (1.4%). All Saudi nationalities patients are covered by the governmental health system, and therefore we do not expect any influence of the health system coverage on medication intake before and during Ramadan in this group.

Since Ramadan lasted from May the 6th until June the 4th and the data were collected in July and August, the time gap could have increased the risk of a recall bias. The study was conducted through face-to-face interviews with multiple interviewers involved in collecting the data in a standardized fashion. Nevertheless, a possible reporting bias could have affected the outcome of the study. The study is also limited by a lack of data on when patients started their anticoagulant therapy, which could have had an influence on their adherence to the medication.

### Summary

Our analysis demonstrated that patient-guided modification of OAC regimen is common during Ramadan. Fasting patients on BID dose encounter a conflict in taking the morning dose as prescribed. This leads to significant changes in OAC intake regimes particularly in BID patients. In consequence, the chance of being admitted to hospital during Ramadan almost triples for patients undertaking self-guided modification of OAC treatment schedules. It is therefore imperative to ensure patients adherence and correct behavior towards OAC intake during Ramadan. Planning the intake of OAC prior to Ramadan and modifying the scheme to suit patient’s fasting times, with special consideration on the time gap between the doses for those on BID medication, is of an essence.

## Electronic supplementary material

Below is the link to the electronic supplementary material.

Supplementary file1 (DOCX 23 kb)
