# Supplementary material for: Patient-guided modifications of oral anticoagulant drug intake during Ramadan fasting: a multicenter cross-sectional study
Source: J Thromb Thrombolysis. 2020 Jul 14;51(2):485–93. doi: 10.1007/s11239-020-02218-0 (PMC7886720; doi:10.1007/s11239-020-02218-0)
Supplement: Supplementary file 1 — Supplementary file1 (DOCX 23 kb) [file 11239_2020_2218_MOESM1_ESM.docx]

Questionnaire:

Patient-guided Modifications of Oral Anticoagulant Drug Intake during Ramadan Fasting: a Multicenter Cross-sectional Study.

All information obtained in this questionnaire will be highly enclosed as anonymous. Personal data of participants will not be declared to any third party. Only the research related data will be studied and published. Participation of the subjects in this study would not affect the provided medical care to them in any way.

Questionnaire’s Number: _ _ _ _ _ _ _ _ _ _ _ _ Date: _ _ _ _ _ _ _ _ _ _ _ _

Center: _ _ _ _ _ _ _ _ _ _ _ _ Outpatient Clincs_ _ _ _ _ _ _ _ _ _ _ _

Personal Informations:

**1- Gender:**  ☐ Male ☐ Female

**2- Age:** _____________________.

**3- Nationality:** ☐ Saudi ☐ Others: Specify _______________.

**4- Religion:** ☐ Muslim ☐ Others: Specify _______________.

**5- Educational Level:** ☐ Bachelor Degree or higher

☐ Less than Bachelor Degree

**6- Career/Job:**  _____________________.

**7- Do you take any Anticoagulation / Blood-thinner medications (for more than 3 Months):**

☐ No ☐ Yes

**8- Have you fasted Ramadan this year:**

☐ No ☐ Yes

Medications’ History in regular times (Before Ramadan):

**9- Why do you take the Anticoagulation / Blood-thinner medications?** (Example: Atrial fibrillation, Heart rhythm disease, etc)

_________________________________________________.

**10- Which Anticoagulation / Blood-thinner medication do you take?**

☐ Dabigatran (Pradaxa®) ☐ Apixaban (Eliquis®)

☐ Rivaroxaban (Xarelto®) ☐ Edoxaban (Lixiana®)

☐ Warfarin (Coumadin®) ☐ Others, specify: _________________.

**11- Please specify the prescribed dose of the Anticoagulation / Blood-thinner?**

Doses:_____________________mg

**12- How many times do you take the Anticoagulation / Blood-thinner medications per day? please specify the time of intake.**

☐ Once daily ☐ Twice daily ☐ Others: _______________

**13- At which time do you take your Anticoagulation / Blood-thinner medications?**

**(In case of twice daily or more, please fill all times of intake seperatly)**

☐ First dose: _______________ ☐ Second Dose: _______________

Medications’ History during Ramadan:

**14- Have your physician changed your medications’ plan so that it suits your fasting in Ramadan ?** ☐ No ☐ Yes

**14a- If Yes, please specify the changes that has been made in your medications’ plan.**

**_________________________________________________________________.**

**15- During Ramadan, what did you do regarding your intake of Anticoagulation / Blood-thinner medications?**

☐ I took my medication on time as prescribed to me from the physician.

☐ I change the medication plan so that it suits my fasting timing.

**16- During Ramadan, have you changed the time of taking your Anticoagulation / Blood-thinner medications without doctor’s consultation different than the prescribed above?**

☐ No ☐ Yes

**17- During Ramadan, were you skipping taking doses of your Anticoagulation / Blood-thinner medications?**

☐ No ☐ Yes

**18- During Ramadan, were you taking or have you took your Anticoagulation / Blood-thinner medications as doubled dose?**

☐ No ☐ Yes

**19- Have you been admitted in a hospital during Ramadan?**

☐ No ☐ Yes

**19a- If Yes, please specify the reason of admission.**

**_________________________________________________________________.**
